# Associations between Sperm Epigenetic Age and Semen Parameters: An Evaluation of Clinical and Non-Clinical Cohorts

**DOI:** 10.3390/cimb46020101

**Published:** 2024-02-16

**Authors:** Savni Sawant, Oladele A. Oluwayiose, Karolina Nowak, DruAnne L. Maxwell, Emily Houle, Amanda L. Paskavitz, Hachem Saddiki, Ricardo P. Bertolla, J. Richard Pilsner

**Affiliations:** 1 C.S. Mott Center for Human Growth and Development, Department of Obstetrics and Gynecology, School of Medicine, Wayne State University, Detroit, MI 48201, USA; savnisawant@wayne.edu (S.S.); ooluwayiose@wayne.edu (O.A.O.); karolinanowak@wayne.edu (K.N.); druanne.maxwell@med.wayne.edu (D.L.M.); ehoule@wayne.edu (E.H.); amanda.paskavitz@med.wayne.edu (A.L.P.); 2 Department of Biochemistry, Microbiology, Immunology, School of Medicine, Wayne State University, Detroit, MI 48201, USA; 3 Department of Environmental Medicine and Public Health, Icahn School of Medicine at Mount Sinai, New York, NY 10029, USA; hachem.saddiki@mssm.edu; 4 Department of Surgery, Division of Urology, Human Reproduction Section, São Paulo Federal University, São Paulo 04024-001, Brazil; rbertolla@gmail.com; 5 Institute of Environmental Health Sciences, Wayne State University, Detroit, MI 48201, USA

**Keywords:** semen analysis, semen parameters, sperm morphology, abnormal morphology, sperm aging, male factor infertility, methylation, pregnancy, sperm epigenetic clock

## Abstract

The well-documented relationship between chronological age and the sperm methylome has allowed for the construction of epigenetic clocks that estimate the biological age of sperm based on DNA methylation, which we previously termed sperm epigenetic age (SEA). Our lab demonstrated that SEA is positively associated with the time taken to achieve pregnancy; however, its relationship with semen parameters is unknown. A total of 379 men from the Longitudinal Investigation of Fertility and Environment (LIFE) study, a non-clinical cohort, and 192 men seeking fertility treatment from the Sperm Environmental Epigenetics and Development Study (SEEDS) were included in the study. Semen analyses were conducted for both cohorts, and SEA was previously generated using a machine learning algorithm and DNA methylation array data. Association analyses were conducted via multivariable linear regression models adjusting for BMI and smoking status. We found that SEA was not associated with standard semen characteristics in SEEDS and LIFE cohorts. However, SEA was significantly associated with higher sperm head length and perimeter, the presence of pyriform and tapered sperm, and lower sperm elongation factor in the LIFE study (*p* < 0.05). Based on our results, SEA is mostly associated with defects in sperm head morphological factors that are less commonly evaluated during male infertility assessments. SEA shows promise to be an independent biomarker of sperm quality to assess male fecundity.

## 1. Introduction

Infertility, or the inability to conceive within 12 months of unprotected intercourse, affects 17.5% of couples around the world [1], and male infertility contributes to nearly half of these cases [2]. An initial evaluation of male infertility includes analyses of semen parameters such as sperm count, concentration, motility, and morphology. Clinically, WHO guidelines for semen analysis serve as the standard for assessing male fertility potential [3]. Over the last 50 years, meta-analyses have indicated significant reductions in sperm concentration [4,5], which has required a continual re-evaluation of semen parameters and what constitutes ‘normal semen’ in updated editions of the WHO Manual for Semen Analysis [6]. Importantly, semen quality measures have been poor predictors of couples’ reproductive outcomes [7,8,9,10,11], suggesting that novel sperm biomarkers of reproductive success are needed [9,12].

While men continuously produce sperm throughout their lifetime, increased paternal age leads to a decline in fertility [13,14] and increases the chances of pregnancy complications, preterm birth, and low birth weight [15]. At a molecular level, the sperm of older men is associated with sperm DNA damage [16], which can occur due to abnormal protamines, oxidative stress, apoptosis, and centrosome aberrations [17]. However, chronological age does not capture intrinsic and extrinsic factors that may contribute to the aging process. As such, the strong association between age and DNA methylation patterns in sperm has permitted the construction of epigenetic clocks to derive an estimate of sperm biological aging [18]. Epigenetic clocks have previously been constructed in somatic cells to predict mortality rates [19,20,21], and we [22] and others [23] have generated sperm-specific clocks to estimate the biological aging of male gametes. Our epigenetic clock, built using a general population cohort, is the first to report associations between sperm epigenetic aging (SEA) and couples’ time-to-pregnancy (TTP), such that men with advanced SEA had lower fecundability and a longer TTP [22]. Subsequently, we have also shown that urinary concentrations of several phthalate metabolites, as well as their mixtures, were associated with higher SEA [24].

To our knowledge the relationship between sperm epigenetic clocks and semen parameters has not been evaluated. To better understand such relationships, we leveraged two cohorts with existing SEA and semen characteristic measurements: the Longitudinal Investigation of Fertility and Environment (LIFE) study is a non-clinical cohort of 379 men, and the Sperm Environmental Epigenetics and Development Study (SEEDS) is a clinical cohort of 192 men seeking fertility treatment. For the LIFE Study, numerous detailed sperm morphological measures were also available for association analyses. Hence, the objective of the current study is to examine the association between SEA and semen parameters by utilizing both clinical and non-clinical cohorts to determine whether the biological aging of sperm is related to semen quality. 

## 2. Methods

### 2.1. Study Populations

To understand the relationships between SEA and semen parameters, we utilized two distinct cohorts. First, the Longitudinal Investigation of Fertility and the Environment (LIFE) study consisted of 501 couples who had stopped contraception to try to become pregnant [7]. The couples were recruited from 16 counties in Michigan and Texas, USA, after browsing local databases. The eligibility criteria for the study included male age ≥18 years; female age <40 years; the ability to communicate in English and/or Spanish; no history of injectable contraceptives in the last 2 years; female menstrual cycles between the range of 21–42 days; and stoppage of contraception within 2 months prior to the start of the study. The research staff systematically acquired data pertaining to population characteristics like age, height and weight, daily habits such as smoking status, and medical histories from both partners. Couples who had been diagnosed with infertility were excluded beforehand. The current study includes 379 (76%) couples who had a remaining aliquot of semen available for DNA methylation analyses. The study participants gave written informed consent before any data collection, and full institutional review board approval was received from all collaborating institutions (Wayne State University Institutional Review Board number 2022 182, November 2022). 

The second cohort was the Sperm Environmental Epigenetics and Development Study (SEEDS), which consisted of 192 couples who were seeking infertility treatment at an IVF clinic at Baystate Medical Center in Springfield, Massachusetts. Eligibility criteria included male participants ≥18 years of age with no history of having a vasectomy and the ability to provide a fresh semen sample following 2–3 days of ejaculatory abstinence. Their female partners had to be below 40 years of age. Clinic staff gathered pertinent information about demographics, lifestyle elements, and medical backgrounds from both partners throughout the IVF cycle. Before collecting samples, each partner filled out an initial questionnaire detailing lifestyle aspects. All study participants provided written informed consent, and ethical approval from the institutional review boards of all collaborating institutions was obtained (Wayne State University Institutional Review Board number 2021 007, February 2021). 

### 2.2. Semen Sample Collection and Measurement of Semen Parameters

In the LIFE cohort, the men were asked to collect semen samples via masturbation at home after at least 2 days of ejaculatory abstinence and without the use of lubricants. The samples were kept on ice overnight and shipped to the laboratory the next day. The laboratory staff then proceeded to conduct a semen analysis manually under a microscope and also input the samples into an HTM-IVOS computer assisted semen analysis (CASA) machine (Hamilton Thorne, Beverly, MA, USA). Along with measuring general semen parameters, detailed sperm morphology was assessed for abnormalities in the sperm head, neck, and tail shape and size [25]. Moreover, DNA integrity parameters such as DNA fragmentation index (DFI) and high DNA stainability (HDS) were measured using the sperm chromatin structural assay (SCSA) [26]. Since semen analysis was conducted after receiving overnight shipments of home-collected semen, motility analyses were excluded from the current analyses. The WHO 2010 cutoffs were the most current at the time and were therefore used to assess the semen parameters. As the LIFE cohort was designed with participants recruited from the general population to identify environmental influences on human fecundity and fertility, detailed semen parameters (e.g., head shapes, morphology, DFI, and HDS) were measured as part of LIFE’s research aims. 

For the SEEDS participants, fresh semen samples were collected at the clinic after a recommended 2–3 day abstinence and were immediately analyzed after 30 min to allow for liquefaction. A basic analysis consisting of sperm count, motility, morphology, volume, and concentration was carried out using guidelines published in the WHO Manual for Semen Processing [25,27]. These analyses also used the WHO 2010 cutoffs. Since this cohort comprises of participants seeking treatment at a fertility clinic, only routinely performed semen analyses for fertility diagnoses before an IVF cycle were performed, and thus, detailed semen parameters for this cohort were not available.

### 2.3. Semen Preparation and DNA Isolation

To isolate sperm from previously aliquoted crude semen from the LIFE cohort, the semen samples were subjected to one-step centrifugation using a 50% density gradient. Sperm from the SEEDS cohort were subjected to gradient centrifugation via a two-step gradient (40% and 80%) at Baystate Medical Center as part of standardized semen processing prior to IVF treatment. Though the initial density gradient centrifugation was carried out differently for both cohorts, DNA extraction and further downstream processing for the EPIC array was conducted with the same protocol for all samples. Since sperm DNA is packaged primarily with protamines instead of histones, sperm needs to be treated with a reducing agent prior to purification. Sperm DNA from both cohorts was extracted using the rapid DNA extraction method developed by our laboratory [28]. Sperm were homogenized with 0.2 mm steel beads and a lysis buffer containing guanidine thiocyanate (Qiagen, Hilden, Germany) and 50 mM tris(2-carboxyethyl) phosphine, a reducing agent, (TCEP; Pierce, Rockford, IL, USA), at room temperature for 5 min. This method consistently gave over 90% high-quality DNA using three different commercially available silica-based spin columns. The protocol offers several advantages: it can be run at room temperature, it does not require lengthy proteinase K (ProK) digestions, and it uses TCEP, which is a stable reducing agent that can be stored at room temperature. This method can be adapted for use in sperm from other mammalian species [29,30]. 

For the LIFE and SEEDS cohorts, sperm DNA methylation analysis was carried out using the EPIC Infinium Methylation Beadchip at the Center for Genome Analyses at Yale University, New Haven, CT, USA (Illumina, San Diego, CA, USA), which covered over 850,000 methylation sites across the genome. To minimalize batch effects, sample randomization was conducted both within and across the beadchip. This was followed by data preprocessing which included normalization of dye bias, batch correction, and the removal of cross-hybridized probes. Quality control and preprocessing resulted in the exclusion of CpG sites with signals that were weak or distorted. Furthermore, as part of our quality control, analyses of *DLK1* and *H19* methylation were conducted to confirm minimal somatic cell contamination of sperm [22].

### 2.4. Sperm Clock Development in the Non-Clinical Cohort

The initial step in constructing an epigenetic clock for sperm involved the identification of individual CpG sites that were significantly associated with male age. As previously described in detail [22], Super Learner [31], an ensemble machine learning technique, was employed to predict biological age and used penalized regressions, such as LASSO, ridge, and elastic net, along with multivariate adaptive regression splines. The age-associated significant CpGs identified were inputted as predictors in Super Learner. These algorithms were used to explore both linear and non-linear relationships between chronological age and the identified significant CpGs. The optimal combination of the algorithm-specific predictions was determined by performing 10-fold cross-validation, which minimized the mean squared error. The performance was assessed using mean absolute error (MAE) and correlations between predicted and chronological age.

Sperm epigenetic age (SEA) was then calculated as the difference between the Super Learner’s predicted age and the chronological age of each participant. This was conducted by performing a linear regression of chronological age versus predicted age, and the deviation of predicted age from chronological age was given as a residual, which was termed ‘SEA’ [22]. Positive SEA values indicated an older epigenetic aging phenotype, while negative values signified a younger epigenetic aging phenotype, as compared to chronological age. For example, an SEA of −5 indicates that the sperm’s predicted age is 5 years lower than the man’s chronological age, whereas an SEA of +5 indicates a predicted sperm age older than the man’s chronological age. Using the information from the CpGs selected by Super Learner, the clock was applied to the SEEDS cohort to derive SEA for those participants.

### 2.5. Statistical Analysis

After visualizing the distributions for the semen parameters with histograms and using the Shapiro–Wilk test, any semen parameters that were not normally distributed were logarithmically transformed to fit linear regression models. Multivariable linear regression between SEA and each individual semen parameter was carried out with R version 4.2.2, adjusting for male smoking status, based on cotinine concentrations, and BMI. 

## 3. Results

### 3.1. Demographics

The demographic and semen parameter characteristics specific to each cohort are presented in Table 1. The mean age for male participants for the LIFE and SEEDS cohorts was 31.9 ± 4.8 years and 36.1 ± 6.5 years, respectively. The majority of the men were non-Hispanic White (81% in the LIFE study and 72% in the SEEDS), non-smokers (79% in the LIFE study and 84% in the SEEDS), and overweight (defined as a BMI of 25–29.9). The majority of the men in the LIFE cohort had a semen profile within the 5% percentile values for fertile men based on the WHO 2010 semen analysis cutoffs. As for the SEEDS cohort, 42.2% of men had sperm morphology below the WHO 2010 cutoff; however, the other semen parameters were generally normal. 

### 3.2. SEA and General Semen Parameters in the LIFE and SEEDS cohorts

**Figure 1:**
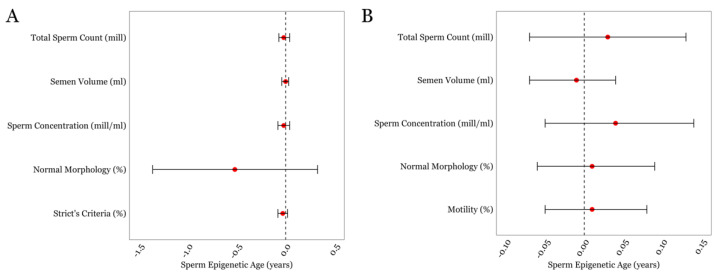
The associations between SEA and routinely measured semen parameters in the LIFE cohort (**A**) and in the SEEDS cohort (**B**). Error bars in the plots represent 95% confidence intervals for the coefficient estimates. Red dots and dotted lines represent the effect estimates for each analysis and the line of null effect, respectively.

We first performed multivariable linear regressions between SEA and general semen parameters in both cohorts (Figure 1), which included semen volume, sperm concentration, total sperm count, and sperm morphology. Sperm motility was included for the SEEDS cohort only as motility assessment for the LIFE cohort was performed after overnight sample shipment. No statistically significant associations were found between the standard semen parameters and SEA within either cohort. The details of the estimates, confidence intervals, and *p*-values for all of the multivariable linear regressions are given in Supplementary Table S1.

### 3.3. SEA and Detailed Semen Parameters in the LIFE cohort

**Figure 2:**
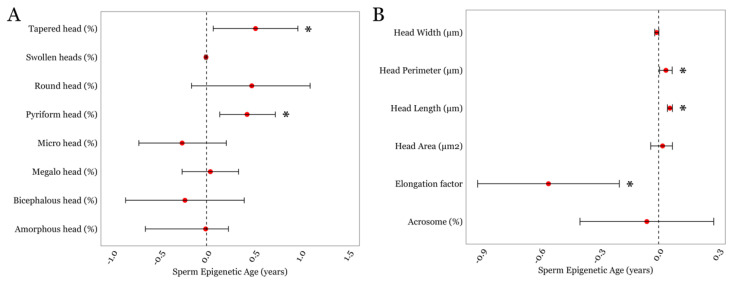
The associations between SEA and detailed sperm head-related morphological parameters in the LIFE cohort. (**A**) Sperm head traits related to its shape and morphometry. (**B**) Sperm head size measurements. * represents a *p*-value of < 0.05. Error bars in the plots represent 95% confidence intervals for the coefficient estimates. Red dots and dotted lines represent the effect estimates for each analysis and the line of null effect, respectively.

Next, we examined associations between SEA and detailed semen parameters that were available in the LIFE cohort. SEA displayed a positive association with two sperm head traits related to shape and morphometry; the presence of tapered sperm (β = 0.52; *p* = 0.02) and the presence of pyriform sperm (β = 0.43; *p* = 0.004); Figure 2A and Supplementary Table S2. We also examined associations between SEA and sperm head size measurements. We observed positive associations between SEA and sperm head perimeter (β = 0.04; *p* = 0.045) and sperm head length (β = 0.03; *p* = 0.008; Figure 2B and Supplementary Table S2). Although SEA and sperm head width displayed an inverse trend, it did not achieve statistical significance (*p* = 0.08). However, we found a significant negative association between SEA and the sperm head elongation factor, expressed as the head’s width-to-length ratio (β = −0.56; *p* = 0.002; Figure 2B and Supplementary Table S2). 

**Figure 3:**
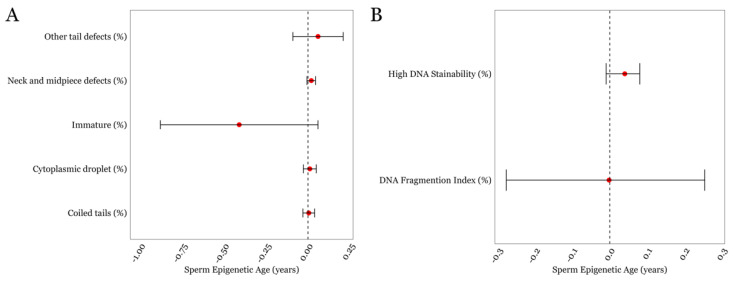
The associations between SEA and various other sperm characteristics in the LIFE cohort. (**A**) Sperm parameters describing regions below the head (**B**) DNA damage-associated parameters. Error bars in the plots represent 95% confidence intervals for the coefficient estimates. Red dots and dotted lines represent the effect estimates for each analysis and the line of null effect, respectively.

Finally, none of the other detailed semen parameters such as neck/midpiece and tail defects exhibited statistically significant associations with SEA. As for the measures related to DNA damage and chromatin structure, the mean ± SD for the DFI and HDS was 15.31 ± 10.4 and 7.4 ± 5.1, respectively, both of which were within the normal range for these parameters, which is <25% [32]. We found that the DFI was not associated with SEA; however, HDS displayed a positive trend with SEA but did not achieve statistical significance (*p* = 0.1; Figure 3B, Supplementary Table S2). 

## 4. Discussion

The current study was conducted to examine the relationship between sperm epigenetic age (SEA) and semen quality, with the objective of providing novel insights into the association between male aging and fertility. While we did not observe significant associations between SEA and routinely analyzed semen parameters such as semen volume, sperm motility, sperm concentration, total sperm count, and normal morphology in either the SEEDS or LIFE cohorts, it did uncover distinctive positive associations between SEA and sperm head morphological traits such as head length, head perimeter, tapered sperm, pyriform sperm, and a negative association between SEA and sperm elongation factor. 

The role of paternal age is often overshadowed by maternal age when considering couples’ fertility potential [33]. Our previous findings showed that advanced SEA was associated with an increased time-to-pregnancy for couples trying to conceive and a shorter gestation time for their offspring, regardless of maternal contributions [22]. Buck Louis et al. in a previous LIFE study observed that the presence of round sperm, amorphous sperm, and sperm with neck-midpiece abnormalities was related to the time-to-pregnancy for couples [34]; however, these semen parameters were not found to be significantly associated with SEA in our study. 

As expected in a clinical fertility cohort, the mean age for the SEEDS participants was five years higher than for the LIFE participants. While the SEEDS participants were older, the range of SEA for the SEEDS participants (−6.0 to 6.7) and the LIFE participants (−4.4 to 5.8) was comparable. It is important to note that SEA was constructed first in the LIFE study and validated in the SEEDS. In fact, predicted age, using the epigenetic clock built in the LIFE study, was strongly correlated with chronological age when applied to SEEDS (r = 0.83; *p* < 2.2 × 10^−16^), indicating that our clock was generalizable in the SEEDS despite having on average older participants. The basic semen parameters in both cohorts were well above the WHO recommended thresholds. However, since the SEEDS is a clinical study involving men seeking fertility treatment, lower values for the semen parameters were expected as compared to the LIFE cohort. Despite the men in the SEEDS study being older and having a higher prevalence of infertility defined by WHO cutoffs, our analyses revealed that SEA was not related to general semen parameters in these two distinct cohorts, indicating that SEA is a molecular signature of sperm aging that is independent of general semen parameters. 

Notably, SEA was not associated with the quality of routine semen parameters, but rather, abnormalities in sperm head morphological parameters that are not typically measured in routine semen analyses. While the exact mechanisms underlying the association between SEA and sperm head parameters require further investigation, this study raises questions about the role of epigenetic changes in early sperm development and subsequent morphology. The negative association between SEA and the sperm head elongation factor indicates that a higher epigenetic age is linked to decreased sperm head elongation, which would cause a blunt and short head shape and could impede the ability of sperm to swim efficiently since the forward propelling motion requires a degree of pointedness in the sperm head [35]. Lower motility, in turn, could decrease the likelihood of sperm successfully reaching and fertilizing the oocyte, causing a longer time to achieve pregnancy. However, it is important to note that SEA was not associated with sperm motility in the SEEDS cohort, and the association between SEA and sperm motility was not conducted in the LIFE study as motility data were only available for sperm after the overnight shipment of semen; and motility is most accurately assessed within 0–2 h of semen collection. 

There is growing interest in enhancing semen analysis by measuring the molecular structure of sperm DNA using tests like the DFI and HDS, which assess DNA strand breaks and retained histones in sperm, respectively. During spermiogenesis, the spermatid nucleus undergoes massive remodeling and condensation, including histone displacement by protamines and disulfide links between protamines to stabilize the nucleus, thereby protecting sperm from external stresses [36]. About 15% of histones are retained in human sperm and an increased histone/protamine ratio has been associated with infertility [37]. Moreover, aberrant histone–protamine exchange reduces the fertilization potential of sperm, which can corelate with abnormal DNA methylation [37,38]. However, while SEA displayed a positive trend with HDS, it did not achieve statistical significance (*p* = 0.1), suggesting a potential relationship with SEA and histone retention. However, histone/protamine ratio data were not available in our two cohorts, and more detailed histone analyses (e.g., specific histone modifications) may be necessary to better assess potential associations. 

Our results also showed that higher SEA is associated with several head morphological abnormalities relating to a larger sperm head phenotype, suggesting incomplete compaction of the sperm nucleus. It is suggested that incomplete histone replacement causes vacuoles to occupy the sperm head and enlarge its shape [39]. It has been established that sperm vacuoles are nuclear concavities that contain DNA and may form from incomplete histone–protamine exchange [40,41]. Indeed, a recent study reported that sperm with vacuoles that occupy more than 15% of the sperm head, compared to normal sperm, were enriched in histone H3 lysine 4 trimethylation (H3K4me3) [39], which has been shown to be an important marker for early-life development [42]. It has been shown that sperm H3K4me3 can be altered due to diet changes, lifestyle, and environmental exposure [43] and can be transmitted to embryos and affect gene expression and development after fertilization [44,45]. This could be due to poor chromatin condensation in enlarged sperm heads, which makes the DNA vulnerable to such unfavorable epigenetic modifications [46]. Since detailed semen parameter assessment was not a research aim in the SEEDS, we could not validate associations between SEA and head defect findings in the LIFE study. 

The integration of epigenetic markers like SEA into fertility assessments has the potential to enhance the precision of diagnosing male infertility, ultimately leading to more personalized and effective treatments for couples struggling with fertility issues. However, additional research is still required to determine the predictive value of SEA on male fecundity. The emergence of omics technologies and the ability to delve into the molecular constituents of sperm offer an avenue for the identification of biomarkers that capture intrinsic facets of male infertility that are susceptible to aging, environmental factors, and lifestyle. Such biomarkers may also extend beyond the epigenetic profiling of sperm DNA, encompassing analyses of seminal protein [9], metabolomics [47,48], and sperm mitochondrial DNA [49]. The identification and validation of such biomarkers hold promise for advancing diagnostic paradigms in male reproductive health, providing a more nuanced understanding of sperm quality, and contributing to enhanced clinical assessments.

We recognize that our study has several strengths and limitations. To our knowledge, this is the first study to elucidate connections between the epigenetic aging of sperm and routine as well as detailed semen parameters. The novelty and potential clinical utility of SEA as a biomarker is underscored by its association with time-to-pregnancy [22] and individual and mixtures of concentrations of urinary phthalate metabolites [24]. An additional strength of our study is the inclusion of two distinct cohorts—comprising clinical and non-clinical populations. Despite these strengths, we recognize that our study also has limitations. First, both the LIFE and SEEDS cohorts predominantly consist of White men; thus, the generalizability of the findings across other ethnic or racial groups is limited. However, it is important to note that male reproductive studies are prone to selection bias with respect to the types of men volunteering to participate [50]. Additionally, the assessment of motility in the samples from the LIFE participants posed a significant challenge due to the samples being kept on ice and shipped overnight to a laboratory. The SEEDS cohort also did not measure detailed sperm parameters such as specific head abnormalities; thus, we were unable to replicate our findings from the LIFE study in our clinical cohort in which a higher incidence of sperm abnormalities would be expected in men seeking infertility treatment. Lastly, the men from the LIFE study could have undiagnosed infertility issues, which may not be captured via semen analyses and highlights the need for novel molecular biomarkers to better assess male fecundity and fertility.

## 5. Conclusions

In summary, while not associated with general semen parameters, SEA was significantly associated with specific sperm head traits. These findings suggest that SEA may be related to abnormalities in the extensive chromatin remodeling during the histone–protamine exchange. SEA assessment offers a distinct fertility biomarker beyond routine analysis, potentially acting as an epigenetic readout of sperm health. Integrating SEA into fertility assessments could provide a more comprehensive understanding of male fertility, although its full practical value in clinical settings requires further exploration.

## Figures and Tables

**Table 1:** Demographic and reproductive characteristics of participants in the LIFE (*n* = 379) and SEEDS (*n* = 192) cohorts.

	LIFE		SEEDS	
	(Mean ± SD or *n* (%))			
Age (years)	31.9 ± 4.8		36.1 ± 6.5	
BMI (kg/m^2^) ^1^	29.9 ± 5.7		29.7 ± 5.5	
Normal (18–25)	67 (18)		38 (20)	
Overweight (>25 <30)	156 (42)		80 (42)	
Obese (>30)	151 (40)		74 (38)	
Race ^1^				
White (non-Hispanic)	307 (81)		139 (72)	
Black (non-Hispanic)	15 (4)		8 (4)	
Hispanic	32 (9)		11 (6)	
Others/Unknown	23 (6)		34 (18)	
Smoking status ^2^				
No	295 (79)		162 (84)	
Yes	80 (21)		30 (16)	
Semen Parameters	Mean ± SD	% < WHO ^3^	Mean ± SD	% < WHO
Sperm count (millions)	74.5 ± 55	7.4	92.9 ± 27.7	4.6
Sperm volume (mL)	3.4 ± 1.7	8.7	2.96 ± 1.4	18.2
Sperm concentration (millions/mL)	228.4 ± 117	7.7	78.4 ± 84.4	9.1
Normal morphology (%)	30 ± 12.2	1.1	6.4 ± 4.5	42.2
Sperm motility (%) ^4^	---		56.9 ± 20.0	9.1
DNA fragmentation index (%) ^5^	15.3 ± 10.4	13.3	---	
High DNA stainability (%) ^5^	7.4 ± 5.1	10.3	---	

^1^ Missing data for *n* = 2 in the LIFE study; ^2^ missing data for *n* = 4 in the LIFE study; ^3^ % of participants below the 2010 WHO reference cutoffs; ^4^ sperm motility not included for the LIFE study; ^5^ the cutoffs for normal DFI and HDS come from standard SCSA protocols [32] and not the WHO 2010 cutoffs.^1^ Missing data for *n* = 2 in the LIFE study; ^2^ missing data for *n* = 4 in the LIFE study; ^3^ % of participants below the 2010 WHO reference cutoffs; ^4^ sperm motility not included for the LIFE study; ^5^ the cutoffs for normal DFI and HDS come from standard SCSA protocols [32] and not the WHO 2010 cutoffs.

## Data Availability

DNA methylation data for the LIFE study are available at the GEO repository at GSE185445; any other data can be made available upon request.

## Supplementary Materials

The following supporting information can be downloaded at: https://www.mdpi.com/article/10.3390/cimb46020101/s1. Table S1: Results of multivariable linear regression models of general semen parameters for the LIFE (*n* = 379) and SEEDS (*n* = 192) cohorts; Table S2: Results of multivariable linear regression models for the detailed semen parameters for the LIFE cohort (*n* = 379).
