# Supplementary material for: Associations between Sperm Epigenetic Age and Semen Parameters: An Evaluation of Clinical and Non-Clinical Cohorts
Source: Curr Issues Mol Biol. 2024 Feb 16;46(2):1567–78. doi: 10.3390/cimb46020101 (PMC10887546; doi:10.3390/cimb46020101)
Supplement: Supplementary file 1 [file cimb-46-00101-s001.zip › cimb-2846515-SI.pdf]

Supplementary Table S1. Results of multivariable linear regression models of general semen parameters for the LIFE (n=379) and SEEDS (n=192) cohorts.

	**LIFE**	**SEEDS**
**Semen Parameter**		
Sperm count (millions)	-0.02 (-0.08, 0.04), 0.57	0.03 (-0.07, 0.13), 0.59
Sperm volume (ml)	-0.001 (-0.04, 0.03), 0.97	-0.01 (-0.07, 0.04), 0.59
Sperm concentration (millions/ml)	-0.02 (-0.07, 0.04), 0.57	0.04 (-0.05, 0.14), 0.39
Normal morphology (%)	-0.52 (-1.36, 0.33), 0.23	0.01 (-0.06, 0.09), 0.69
Sperm motility (%)	---	0.01 (-0.05, 0.08), 0.74
Strict's Criteria (%)	-0.03 (-0.08, 0.03), 0.19	---

Models were adjusted for male BMI and smoking status. Data presented as β-estimate (95% confidence interval), p-value.

Supplementary Table S2. Results of multivariable linear regression for the detailed sperm parameters for the LIFE cohort (n=379).

Detailed Sperm Parameters	β-estimate (95% CI), p-value
**Head Descriptive Parameters**	
Head Width (μm)	-0.01 (-0.02, 0.001), 0.08
Head Perimeter (μm)	**0.04 (0.01, 0.07), 0.045 ***
Head Length (μm)	**0.03 (0.07, 0.05), 0.008 ***
Head Area (μm^2)	0.02 (-0.04, 0.07), 0.56
Elongation factor	**-0.56 (-0.92, -0.2), 0.002 ***
Acrosome (%)	-0.06 (-0.4, 0.28), 0.72
**Head Morphology parameters (%)**	
Tapered head	**0.52 (0.07, 0.97), 0.02 ***
Swollen heads	-0.01 (-0.02, 0.004), 0.22
Round head	0.48 (-0.16, 1.1), 0.14
Pyriform head	**0.43 (0.14, 0.73), 0.004 ***
Micro head	-0.26 (-0.72, 0.21), 0.29
Megalo head	0.04 (-0.26, 0.34), 0.79
Bicephalous head	-0.23 (-0.86, 0.40), 0.47
Amorphous head	-0.01 (-0.65, 0.23), 0.35
**Miscellaneous abnormalities (%)**	
Other tail defects	0.06 (-0.09, 0.21), 0.42
Neck and Midpiece defects	0.02 (-0.006, 0.05), 0.14
Immature	-0.41 (-0.88, 0.06), 0.09
Cytoplasmic droplet	0.01 (-0.03, 0.05), 0.58
Coiled tails	0.01 (-0.03, 0.04), 0.75
**Sperm DNA Integrity (%)**	
High DNA Stainability	0.04 (-0.01, 0.08), 0.1
DNA Fragmentation Index	-0.002 (-0.28, 0.25), 0.93

Models were adjusted for male BMI and smoking status. Data presented as β-estimate (95% confidence interval), p-value. * represents a p-value of < 0.05.
